# Photodistributed exfoliative dermatitis associated with semaglutide: Expanding the spectrum of glucagon-like peptide-1 receptor agonist–associated cutaneous reactions

**DOI:** 10.1016/j.jdcr.2026.03.062

**Published:** 2026-04-09

**Authors:** Rebecca Kann, Yael Weitzner, Cyrene Tan, Cynthia X. Chan, Bijal Amin, Shira Wieder

**Affiliations:** a Division of Dermatology, Albert Einstein College of Medicine, Montefiore Medical Center, Bronx, New York; b Department of Pathology, Albert Einstein College of Medicine, Montefiore Medical Center, Bronx, New York

**Keywords:** drug reaction, GLP-1 agonists, semaglutide

## Introduction

Semaglutide, a glucagon-like peptide-1 receptor agonist (GLP-1 RA), is an increasingly popular medication for improving glycemic control and promoting weight loss in patients with type 2 diabetes or obesity.^1^ Although gastrointestinal symptoms are the most commonly reported adverse events, cutaneous reactions are less frequently described.^2^ Rare but significant eruptions have been reported, with morphologies including dermal, bullous, and vasculitic hypersensitivity reactions; morbilliform, psoriasiform, and fixed drug eruptions; and eosinophilic panniculitis.^3^ Early recognition is critical, as prompt identification and discontinuation of semaglutide often leads to rapid resolution of cutaneous findings.^4^ We report a case of photodistributed, exfoliative dermatitis temporally associated with semaglutide initiation.

## Case report

**Fig 1:**
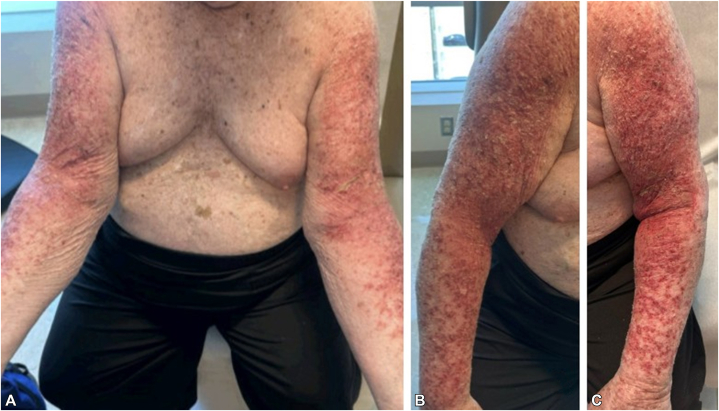
Photodistributed, erythematous, scaly plaques on the **(A)** flexor aspects, including the antecubital fossae, and the extensor surfaces of the **(B)** right and **(C)** left upper extremities.

A 77-year-old man presented with a pruritic, erythematous, scaly rash affecting both upper extremities. The eruption initially involved the extensor aspects of bilateral arms and was mistaken by the patient for sun damage after sun exposure; it progressively expanded to involve the flexor surfaces, including the antecubital fossa. Examination revealed exfoliative, erythematous plaques on the upper extremities (Fig 1, *A-C*).

Past dermatologic history included squamous cell carcinoma, basal cell carcinoma, and keratoacanthoma, each previously treated with Mohs micrographic surgery, as well as multiple actinic keratoses managed with cryotherapy and 5-fluorouracil. Medical history was notable for obesity (body mass index, 39.9 kg/m^2^), hypertension, hyperlipidemia, chronic obstructive pulmonary disease, and prior venous thromboembolism. Medications included apixaban, atorvastatin, famotidine, lisinopril, oxybutynin, sertraline, tamsulosin, and zolpidem. Semaglutide (Ozempic) was initiated as a 0.25 mg subcutaneous injection 2 months prior to rash onset, with dose escalation to 0.50 mg occurring days before rash development. Prior to biopsy of the rash, the differential diagnosis included actinic drug eruption, severe contact dermatitis, and psoriasis.

**Fig 2:**
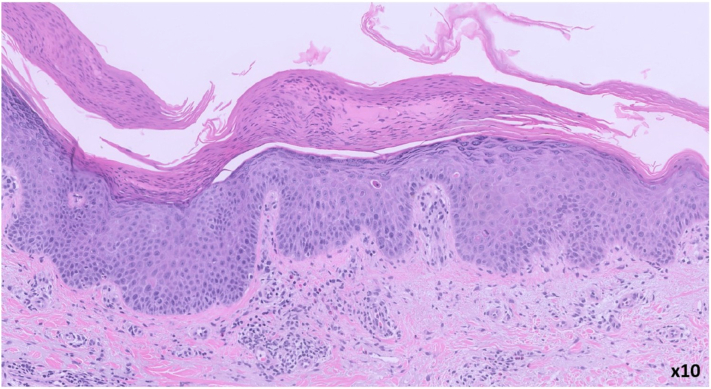
The epidermis is parakeratotic, hyperplastic, and slightly spongiotic, with several dyskeratotic keratinocytes. There is focal, subtle vacuolar interface dermatitis, as well as superficial perivascular lymphocytic inflammation with purpura and eosinophils. (Hematoxylin and eosin stain; original magnification: ×10.)

**Fig 3:**
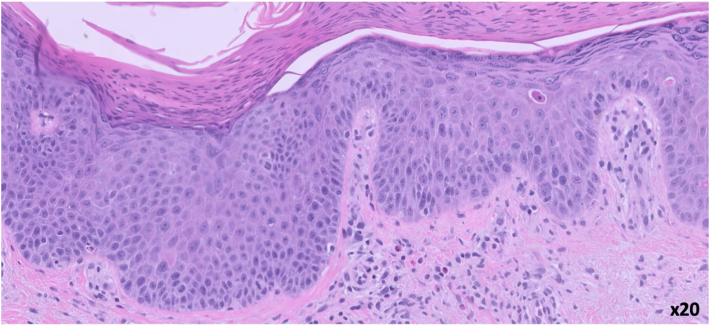
There are suprabasilar dyskeratotic keratinocytes and superficial perivascular lymphocytes and eosinophils. (Hematoxylin and eosin stain; original magnification: ×20).

A shave biopsy taken from the left arm demonstrated a spongiotic, psoriasiform, and interface dermatitis with eosinophils and purpura (Figs 2 and 3). The presence of numerous eosinophils and subtle interface changes was suggestive of a drug-associated process.

**Fig 4:**
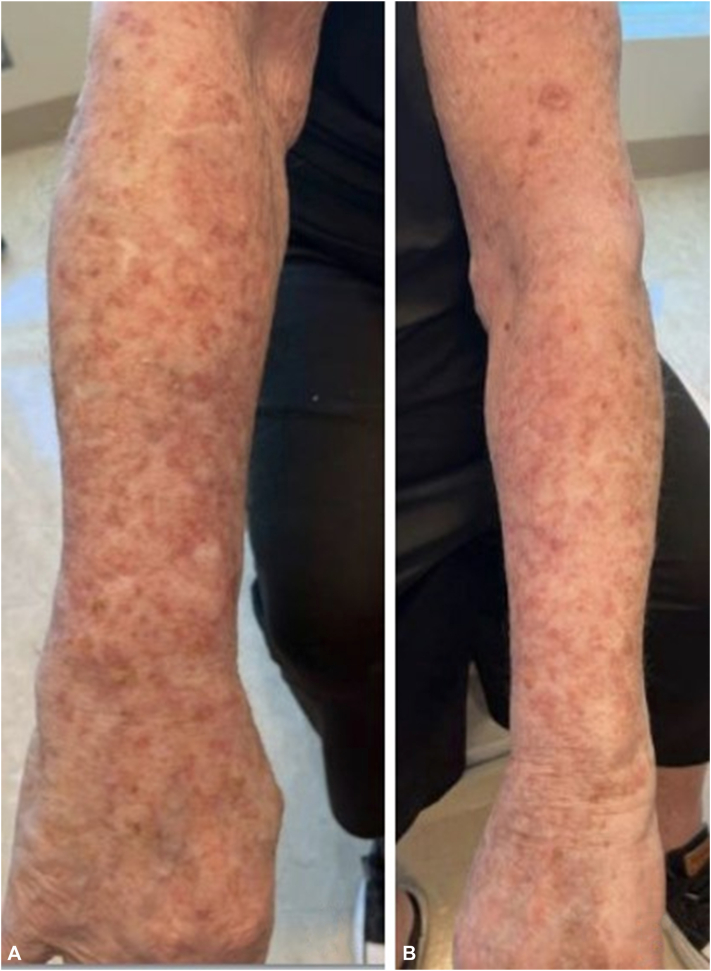
Resolution of lesions on the **(A)** right and **(B)** left arms 1 month after discontinuation of semaglutide with application of fluocinonide ointment 0.05%. Some remaining postinflammatory erythema and residual actinic damage is present.

The patient discontinued semaglutide and applied fluocinonide 0.05% ointment twice daily for 14 days. Within 1 week, he reported near-complete resolution of the eruption (Fig 4).

The temporal relationship, histopathologic findings, and rapid improvement following drug cessation supported a diagnosis of semaglutide-associated photodermatitis.

## Discussion

GLP-1 RAs, including semaglutide, liraglutide, and dulaglutide, are increasingly prescribed for type 2 diabetes mellitus and obesity due to their glycemic and cardiometabolic benefits. Although their safety profile is favorable, cutaneous adverse events are increasingly recognized, most commonly eczematous eruptions, pruritus, drug exanthems, hyperhidrosis, and alopecia.^5^

Reported cutaneous adverse reactions to GLP-1 receptor agonists often demonstrate a delayed onset, most commonly occurring weeks to months after drug initiation.^3^ This case describes a delayed cutaneous reaction likely resulting from re-exposure to the drug with dose escalation after a period of immunologic sensitization. Alternative explanations for this patient’s cutaneous reaction include viral exanthems, allergic contact dermatitis, and primary eczematous dermatitis. However, clinical evaluation and history did not reveal viral symptoms, new exposures, or environmental factors suggestive of these conditions.

In this case, the clinical and histopathologic findings were most consistent with drug-associated dermatitis. GLP-1 RAs modulate multiple immune pathways, including T-helper 1/T-helper 17 and T-helper 2 cytokine signaling, potentially altering keratinocyte proliferation and cytokine release.^6^ Paradoxically, although several studies report improvement in psoriasis and other inflammatory dermatoses with GLP-1 RAs, others describe new-onset or exacerbated psoriasiform reactions.^6^^,^^7^

Although morbilliform drug eruptions have been described with GLP-1 RAs,^8^ our case is distinguished by its purpuric morphology and mixed spongiotic–psoriasiform–interface pattern with eosinophils. These features expand the known spectrum of GLP-1–associated eruptions.

Clinically, establishing a temporal association between drug exposure and eruption onset, coupled with rapid resolution after drug discontinuation, is key to the diagnosis of cutaneous drug reactions. In this patient, cessation of semaglutide and initiation of topical corticosteroids were sufficient for complete resolution. Given the therapeutic benefit and limited alternatives to GLP-1 receptor agonists, a treat-through approach may be reasonable for mild, non–life-threatening cutaneous eruptions, using topical corticosteroids and close monitoring. Through shared decision-making, the patient elected to discontinue the medication given the extent of cutaneous involvement. Management should be individualized based on patient comorbidities and the extent of disease.

This case contributes to the growing body of literature on GLP-1 RA–associated dermatoses, highlighting the need for clinician awareness as these agents gain broader indications. Early recognition facilitates timely resolution and prevents unnecessary systemic therapy for presumed primary inflammatory dermatoses.

## Conflict of interest

None disclosed.
